# Structural insights into the recognition of phosphorylated FUNDC1 by LC3B in mitophagy

**DOI:** 10.1007/s13238-016-0328-8

**Published:** 2016-10-18

**Authors:** Mengqi Lv, Chongyuan Wang, Fudong Li, Junhui Peng, Bin Wen, Qingguo Gong, Yunyu Shi, Yajun Tang

**Affiliations:** Hefei National Laboratory for Physical Sciences at Microscale and School of Life Sciences, University of Science and Technology of China, Hefei, 230027 China

**Keywords:** microtubule-associated protein light chain 3 beta (LC3B), fun14 domain-containing protein 1 (FUNDC1), phosphorylation, selective mitophagy

## Abstract

Mitophagy is an essential intracellular process that eliminates dysfunctional mitochondria and maintains cellular homeostasis. Mitophagy is regulated by the post-translational modification of mitophagy receptors. Fun14 domain-containing protein 1 (FUNDC1) was reported to be a new receptor for hypoxia-induced mitophagy in mammalian cells and interact with microtubule-associated protein light chain 3 beta (LC3B) through its LC3 interaction region (LIR). Moreover, the phosphorylation modification of FUNDC1 affects its binding affinity for LC3B and regulates selective mitophagy. However, the structural basis of this regulation mechanism remains unclear. Here, we present the crystal structure of LC3B in complex with a FUNDC1 LIR peptide phosphorylated at Ser17 (pS_17_), demonstrating the key residues of LC3B for the specific recognition of the phosphorylated or dephosphorylated FUNDC1. Intriguingly, the side chain of LC3B Lys49 shifts remarkably and forms a hydrogen bond and electrostatic interaction with the phosphate group of FUNDC1 pS_17_. Alternatively, phosphorylated Tyr18 (pY_18_) and Ser13 (pS_13_) in FUNDC1 significantly obstruct their interaction with the hydrophobic pocket and Arg10 of LC3B, respectively. Structural observations are further validated by mutation and isothermal titration calorimetry (ITC) assays. Therefore, our structural and biochemical results reveal a working model for the specific recognition of FUNDC1 by LC3B and imply that the reversible phosphorylation modification of mitophagy receptors may be a switch for selective mitophagy.

**Electronic supplementary material:**

The online version of this article (doi:10.1007/s13238-016-0328-8) contains supplementary material, which is available to authorized users.

## Introduction

As the source of ATP, mitochondria play a central role in cellular metabolism, stress responses and cell death (Galluzzi et al., 2012). Mitochondrial dysfunction results in a large number of reactive oxygen species (ROS), which damage the mitochondrial and cellular DNA and proteins (Lemasters, 2005; Wallace, 2005; Murphy, 2013). The quality control of mitochondria is crucial for the normal physiological function of cells (Rugarli and Langer, 2012; Javadov and Kuznetsov, 2013). Cells employ mitophagy as a major strategy to eliminate the dysfunctional mitochondria produced under stress (Batlevi and La Spada, 2011; Kanki et al., 2011). The main process of mitophagy in mammals is lysosome-dependent and includes the selective removal of abnormal mitochondria with low membrane potential (Bampton et al., 2005; Levine and Kroemer, 2008; Narendra et al., 2008). A considerable number of studies suggested that mitophagy is regulated through two pathways, including receptor-mediated mitophagy and Parkin-dependent mitophagy (Kim et al., 2007; Narendra et al., 2008; Okamoto et al., 2009; Novak et al., 2010; Liu et al., 2012). Many neurodegenerative diseases, such as Parkinson’s disease and Alzheimer’s disease, and cancers, such as hepatocellular carcinoma, are related to the dysfunction of mitophagy (Chu et al., 2007; Mizushima et al., 2008; Dikic et al., 2010; Deas et al., 2011; Ding et al., 2011).

Recently, several mitophagy receptors have been identified, such as ATG32 in yeast (Kanki et al., 2009; Okamoto et al., 2009), and NIX/BNIP3L (Novak et al., 2010), BNIP3 (Hanna et al., 2012), FUNDC1 (Liu et al., 2012) in mammalian cells. One of the common characteristics of mammalian mitophagy receptors is their conserved LC3 interaction region (LIR) with a W/Y/FxxL/I/V motif, which can interact with LC3, a bio-marker protein of the autophagosome in mammalian cells (Pankiv et al., 2007; Noda et al., 2008; Novak et al., 2010; Liu et al., 2012). The post-translational modifications of the mitophagy receptors, especially phosphorylation, play the key roles in regulating mitochondria homeostasis (Egan et al., 2011; Liu et al., 2014). In line with these studies, the phosphorylation of ATG32 promotes its interactions with both ATG11 and ATG8 (Farre et al., 2013) and subsequently triggers mitophagy. Moreover, the selective mitophagy receptor BNIP3 enhances its interaction with LC3B and causes an increased mitophagy through the serine phosphorylation of LIR (Zhu et al., 2013).

**Fig. 1:**
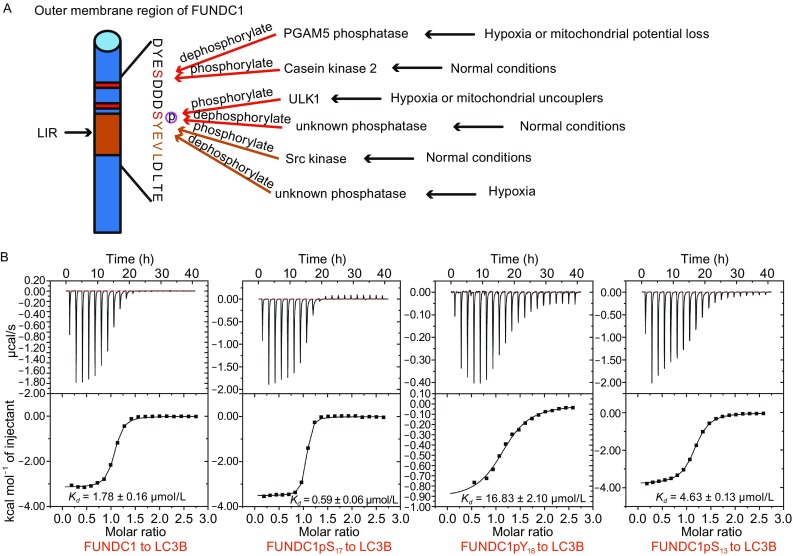
**The signaling pathways of FUNDC1-mediated mitophagy are regulated by phosphorylation modification**. (A) Schematic representation of reversible phosphorylation at critical sites in the FUNDC1 mitochondrial outer membrane region. (B) The ITC fitting results of LC3B with unphosphorylated FUNDC1 peptide and FUNDC1 peptides phosphorylated at different positions

As a lately reported receptor for hypoxia-induced mitophagy in mammalian cells, FUNDC1, which is localized at the outer membrane of mammalian mitochondria, forms a high hydrophobicity transmembrane domain with three-helices and exposes its N-terminus to the cytoplasm (Liu et al., 2012). Interestingly, several reports have revealed that the outer membrane region of FUNDC1 interacts with LC3B and regulates hypoxia-induced selective mitophagy through the reversible phosphorylation at several critical sites (Liu et al., 2012; Chen et al., 2014; Wu et al., 2014). As previously reported, FUNDC1 interacts with LC3B through its classical LIR - Y^18^xxL^21^, while the phosphorylation at Tyr18 by Src kinase remarkably reduces the FUNDC1-mediated mitophagy. Under hypoxic stress, Tyr18 is dephosphorylated to promote the interaction between FUNDC1 and LC3B and mitophagy is triggered (Liu et al., 2012). In this context, Ser/Thr kinase CK2 phosphorylates the Ser13 of FUNDC1 in normal cells, while PGAM5 phosphatase in mitochondria dephosphorylates Ser13 under hypoxia stimulation. The dephosphorylation of Ser13 results in the enhanced interaction of FUNDC1 with LC3B, which further leads to the selective removal of dysfunctional mitochondria (Chen et al., 2014). On the other hand, Ser17 of FUNDC1 is phosphorylated by ULK1 kinase under hypoxia or mitochondrial uncouplers stimulation to increase the binding affinity for LC3B and promote mitophagy (Wu et al., 2014) (Fig. 1A). Although various intracellular experiments have confirmed that the reversible phosphorylation of FUNDC1 plays the key role in the regulation of mitophagy (Liu et al., 2012; Chen et al., 2014; Wu et al., 2014), the precise working mechanism remains unclear and needs to be elucidated.

To gain structural insights into the interactions between FUNDC1 and LC3B involved in hypoxia-induced selective mitophagy, it is essential to solve the structure of LC3B in complex with FUNDC1, including LIR and phosphorylated critical residues, at atomic resolution. Although the apo LC3B structure and a set of LC3B-LIR complex structures have been reported (Ichimura et al., 2008; Rogov et al., 2013; Suzuki et al., 2014; McEwan et al., 2015), the structural basis for the phosphorylation-regulated interaction between FUNDC1 LIR peptide and LC3B is still largely unknown. Here, we present the crystal structure of LC3B in complex with a FUNDC1 LIR peptide phosphorylated at Ser17 (pS_17_). Through the structural analyses, we identified the key residues of LC3B responsible for the specific recognition of the phosphorylated or dephosphorylated FUNDC1. We found that the phosphate group of FUNDC1 pS_17_ binds to LC3B Lys49 and enhances the binding affinity, while the phosphorylation of FUNDC1 Tyr18 may conflicts with the hydrophobic pocket of LC3B and disrupt the interaction. In addition, using the High Ambiguity Driven protein-protein Docking (HADDOCK) and ITC, we were able to show that LC3B Arg10 interacts with FUNDC1 Ser13, but the phosphorylation of FUNDC1 Ser13 may generate steric hindrance for LC3B binding. Furthermore, mutation and ITC assays were performed to validate our observations from the crystal structure. Our structural and *in vitro* interaction analyses provide a detailed elucidation of the specific recognition of FUNDC1 by LC3B and facilitate a deep understanding of how mitophagy receptors utilize the post-translational modification to sense environmental stress and elaborately regulate the selective mitophagy.

## Results

### The interaction between LC3B and FUNDC1 is dramatically affected by the phosphorylation states of FUNDC1

Three key residues, Ser13, Ser17 and Tyr18, in the outer membrane region of FUNDC1 and their phosphorylation states have been reported to play essential roles in affecting the binding affinity for LC3B and influence the FUNDC1-mediated selective mitophagy (Liu et al., 2012; Chen et al., 2014; Wu et al., 2014). In line with these results, a series of FUNDC1 peptides were first synthesized and tested for their binding affinities for LC3B. These FUNDC1 peptides all include LIR and flanking residues (10–25: DYESDDDSYEVLDLTE), with/without the phosphorylation at different positions. To investigate the LC3B-FUNDC1 interaction quantitatively, we employed ITC to measure the binding affinities of LC3B to the FUNDC1 peptides. First, we measured the binding affinity of LC3B to the FUNDC1 peptide without any phosphorylation modification and the *K*_*D*_ value was fitted to 1.78 ± 0.16 μmol/L. We then measured the binding affinity of LC3B with three peptides containing pS_13_, pS_17_ and pY_18_, respectively. The ITC results show that FUNDC1 pS_17_ peptide binds to LC3B ~3-fold stronger than the unphosphorylated FUNDC1 peptide, while the FUNDC1 pS_13_ and FUNDC1 pY_18_ peptides bind to LC3B ~3-fold and ~10-fold weaker than the unphosphorylated peptide, respectively (Fig. 1B). Taken together, our results demonstrate that phosphorylation of FUNDC1 Ser17 enhances the interaction between FUNDC1 and LC3B, while the phosphorylation of FUNDC1 Ser13 and Tyr18 reduce the binding affinities, consistent with the previously reported experiment results *in vivo*.(Liu et al., 2012; Chen et al., 2014; Wu et al., 2014)

### Overall structure of the LC3B-FUNDC1 pS_17_ peptide complex

To provide structural insights into the interaction between LC3B and FUNDC1, X-ray crystallography was employed to study the complex structure. We chose a FUNDC1 LIR peptide^10–25^ with pS_17_, which simulates the possible physiological state upon the induction of mitophagy and exhibits a strong binding affinity for LC3B, to co-crystallize with full-length LC3B^1–125^. The crystal structure of the LC3B-FUNDC1 complex was subsequently refined to a resolution of 2.25 Å in space group P 2_1_. The crystal structure was solved by molecular replacement using the structure of apo LC3B (PDB ID: 3VTU) as the search model. Finally, the R_work_ and R_free_ of the LC3B-FUNDC1 complex structure were refined to 20.57% and 27.22%, respectively. The detailed crystallographic statistics are summarized in Table 1.

**Table 1:** Data collection and refinement statistics

Data collection	LC3B-FUNDC1 pS_17_
Space group	P2_1_
Wavelength (Å)	0.9792
Resolution (Å)	37.88–2.25 (2.33–2.25)
Cell dimensions	
a, b, c (Å)	40.54, 86.85, 40.54
α, β, γ (°)	90.00, 110.86, 90.00
Unique reflections	12034 (1167)
Completeness (%)	96.8
I/σI	7.5 (3.8)
R_merge_ (%)	7.6 (31.4)
R_meas_ (%)	9.8 (40.6)
CC1/2^b^	0.988
Refinement	
R_work_ (%)	20.57
R_free_ (%)	27.22
Average B factors (Å^2^)	
Protein	33.46
H_2_O	34.77
Root mean square deviations	
Bond lengths (Å)	0.011
Bond angles (°)	1.450
Ramachandran plot	
Favored (%)	99.2
Allowed (%)	0.8
Disallowed	0

**Fig. 2:**
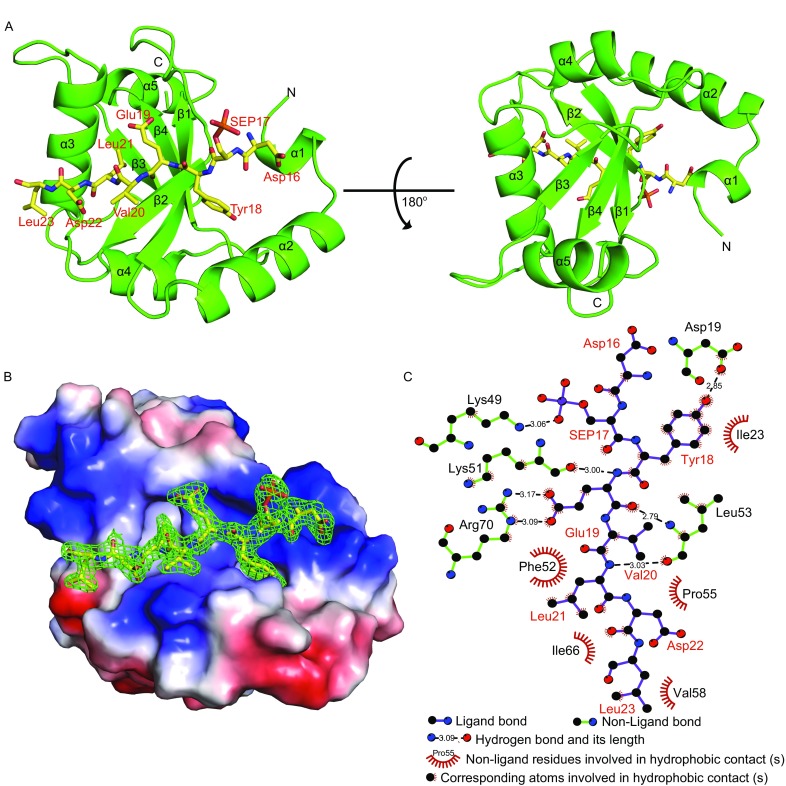
**Crystal structure of LC3B-FUNDC1 pS**_**17**_ **peptide complex**. (A) Cartoon representation of LC3B (green) in complex with the FUNDC1 pS_17_ peptide (yellow). In the peptide, the phosphorylated Ser17 is labeled as SEP17. (B) The electrostatic potential of the LC3B-FUNDC1 pS_17_ peptide complex surface, in which positively charged, negatively charged and neutral areas are represented in blue, red and white, respectively. The 2Fo-Fc omit density map of the FUNDC1 pS_17_ peptide was contoured at 1.0 σ (green). (C) Schematic representations of the recognition of FUNDC1 pS_17_ peptide (colored purple and labeled in red) by LC3B (colored green and labeled in black)

In the final model, the LC3B-FUNDC1 complex includes two molecules in an asymmetric unit both with observable electronic densities for 120 amino acid residues of LC3B^5–124^ and a main part of the FUNDC1 pS_17_ peptide^16–23^ (D-pS_17_-YEVLDL). The two molecules display a root-mean-square (r.m.s.) deviation for Cα atoms of 0.248 Å, revealing only slight conformational differences between the two molecules in the flexible loops. The overall structure of the LC3B-FUNDC1 complex is depicted in Fig. 2A and 2B. The LC3B in complex with FUNDC1 exhibits four-stranded anti-parallel β sheets (β1–β4) separated by five α-helices (α1–α5) which is highly similar to the structure of apo LC3B (Rogov et al., 2013) (with r.m.s. deviations for Cα atoms of 0.695 Å). The FUNDC1 pS_17_ peptide binds to one side of the LC3B surface and lies on the β2 of LC3B in an extended conformation (Fig. 2A).

**Fig. 3:**
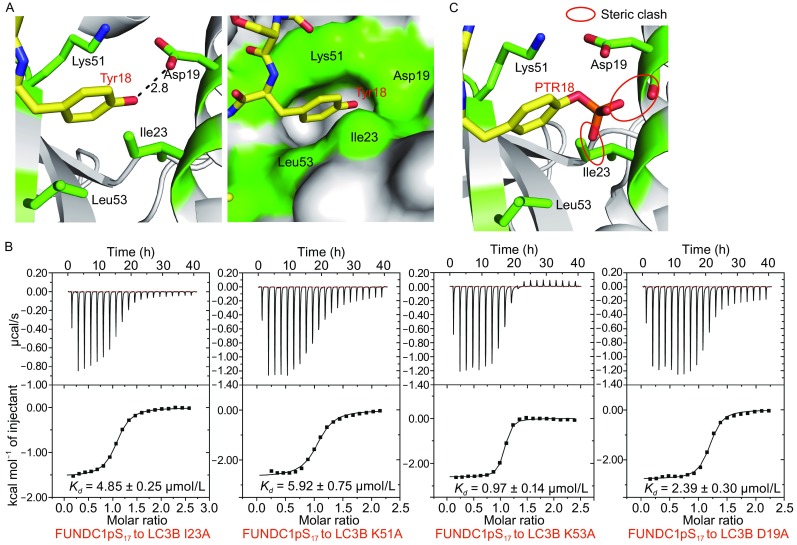
**The phosphorylation of FUNDC1 Tyr18 disrupts the interaction with the hydrophobic pocket of LC3B**. (A) FUNDC1 Tyr18 inserts into the deep hydrophobic pocket of LC3B and forms a hydrogen bond with LC3B Asp19. The hydrogen bond is indicated as a black dash. The surrounding structure of LC3B is represented in grey. FUNDC1 Tyr18 is colored yellow and labeled in red. The residues of LC3B involved in the interaction with FUNDC1 Tyr18 (Asp19, Ile23, Lys51 and Leu53) are colored green and labeled in black. (B) The ITC fitting results of the FUNDC1 pS_17_ peptide with LC3B mutated at the above-mentioned positions. (C) The hypothetical model of PTR18 (phosphorylated Tyr18, phosphorylated by PyTMs) in the complex structure. The steric clash is highlighted with red ovals

The interactions between LC3B and FUNDC1 pS_17_ peptide^16–23^ are shown in Fig. 2C, including hydrogen bonding interactions and hydrophobic interactions. The side chains of FUNDC1 Tyr18 and Leu21 insert into two deep hydrophobic pockets of LC3B, resulting in specific recognitions between LC3B and FUNDC1 (Fig. 2A and 2B). The pocket accommodating the side chain of Tyr18 is mainly composed of several hydrophobic residues including Ile23, Lys51 and Leu53 of LC3B, and forms a hydrogen bond between the carbonyl oxygen of LC3B Asp19 and the hydroxyl group of FUNDC1 Tyr18 (Fig. 3A). To validate the importance of these conserved residues of LC3B involved in FUNDC1 Tyr18 recognition, we introduced alanine mutations into these residues to partially disrupt the hydrophobic pocket and performed ITC assays to measure their binding affinities for the FUNDC1 pS_17_ peptide. As shown in Table 2, all LC3B mutants weaken the interactions of LC3B with the FUNDC1 pS_17_ peptide, particularly I23A and K51A, which exhibit more than 5-fold reductions in binding affinities (*K*_*D*_ = 4.85 ± 0.25 μmol/L and 5.92 ± 0.75 μmol/L, respectively), compared with 0.59 ± 0.06 μmol/L of wild-type LC3B (Fig. 3B). Similar to the mutations effect on the hydrophobic pocket of LC3B, the phosphorylation of FUNDC1 Tyr18 (PTR18) leads to a more extended side chain, which may conflict with the hydrophobic pocket and disrupt the interaction (Fig. 3C).

**Table 2:** The thermodynamic parameters of the ITC experiments

Peptide	LC3B	ΔH kcal/mol	ΔS cal/mol/K	*K*_*D*_ μmol/L	N
FUNDC1	Wild type	−3.15	15.70	1.78 ± 0.16	1.02
	R10A	−3.56	10.70	11.44 ± 1.14	1.01
	R11A	−4.51	8.90	5.62 ± 0.25	1.12
	F7A	−3.08	14.60	3.52 ± 0.23	1.17
	V46A	−4.86	10.20	1.59 ± 0.19	1.02
	L47A	−2.06	18.3	3.07 ± 0.64	1.19
	D48A	−3.03	17.00	1.17 ± 0.15	0.98
	T50A	−2.65	16.50	2.81 ± 0.31	1.02
FUNDC1 pS_13_	Wild type	−3.81	11.60	4.63 ± 0.13	1.13
	R10A	−3.67	8.47	28.49 ± 2.97	1.02
	R10E	−2.83	11.00	34.01 ± 3.46	1.03
	R11A	−4.10	8.91	11.17 ± 0.73	1.11
	R11E	−2.39	13.80	17.12 ± 1.05	1.04
FUNDC1 pS_17_	Wild type	−3.51	16.7	0.59 ± 0.06	1.00
	K49A	−4.90	8.5	3.56 ± 0.21	1.08
	K49E	−4.49	2.87	120.48 ± 5.72	0.93
	K49R	−5.78	10.2	0.33 ± 0.07	1.17
	R70A	−1.80	15.9	15.75 ± 1.50	1.04
	R70E	−2.54	10.8	60.98 ± 3.44	1.03
	D19A	−2.78	16.4	2.39 ± 0.30	1.16
	I23A	−1.53	19.2	4.85 ± 0.25	1.04
	K51A	−2.66	15.0	5.92 ± 0.75	1.03
	L53A	−2.61	18.7	0.97 ± 0.14	1.03
FUNDC1 pY_18_	Wild type	−0.93	18.7	16.83 ± 2.10	1.16

**Fig. 4:**
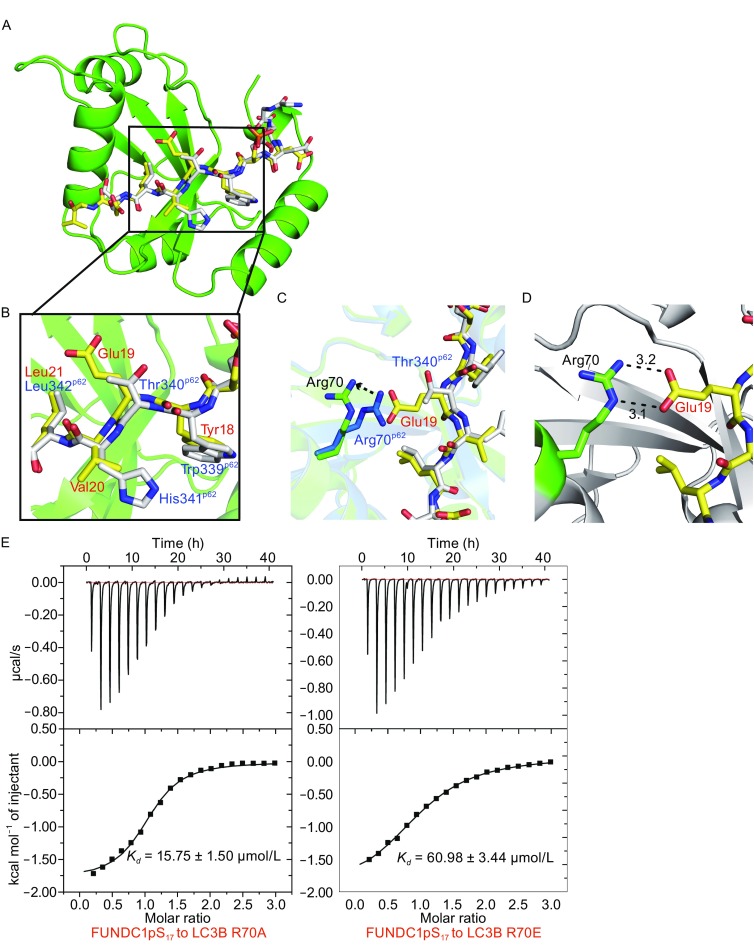
**Comparison of the LC3B-FUNDC1 complex and LC3B-p62 complex**. (A) The superimposition of the LC3B-FUNDC1 complex on the LC3B-p62 complex. The superimposition only shows LC3B (green) in the LC3B-FUNDC1 complex (in two complexes, the two LC3B molecules only display a r.m.s. deviation for Cα atoms of 0.489 Å). The FUNDC1 peptide is shown in yellow while the p62 peptide is colored white. (B) Close-up view of the structural comparison of LIRs in FUNDC1 (yellow) and p62 (white). Residues of LIRs in FUNDC1 and p62 are labeled in red and blue, respectively. (C) The difference of LC3B Arg70 (green and blue, respectively) recognized by FUNDC1 Glu19 (yellow) and p62 Thr340 (white). (D) Interactions between LC3B Arg70 and FUNDC1 Glu19. The surrounding structure of LC3B is represented in grey. Salt bonds are indicated as black dashes. (E) The ITC fitting results of FUNDC1 pS_17_ peptide with LC3B mutants at the Arg70 position

The interaction pattern between LC3B and FUNDC1 LIR is quite similar to those between LC3B and other autophagy-related LIR in complex structures (Figs. 4A and Fig. S1). Likewise, the LC3B residues involved in hydrophobic interactions with different W/Y/FxxL/I/V LIR motifs are identical (Figs. 4A and S1). To further compare our LC3B-FUNDC1 complex with other LC3B-autophagy receptor complexes, we superimposed our complex structure on the LC3B-p62 complex (PDB code 2ZJD), which is involved in the ubiquitin-dependent selective autophagy (Ichimura et al., 2008) (Fig. 4A). LC3B and the peptides in the two complexes share highly similar conformations, respectively. Both YxxL motif of FUNDC1 and WxxL motif of p62 exhibit a residue with a bulky aromatic side chain (Y or W), which deeply insert into the hydrophobic pockets of LC3B, sharing the similar interactions (Fig. 4A and 4B). Nevertheless, Glu19 in the FUNDC1 LIR forms a distinct interaction with LC3B Arg70 from its corresponding residue in p62, Thr340 (Fig. 4B and 4C). As shown in Fig. 4C, the side chain of FUNDC1 Glu19 is longer than p62 Thr340, posing a steric clash with LC3B Arg70 to induce the torsion of Arg70 side chain. Moreover, unlike p62 Thr340, FUNDC1 Glu19 forms additional salt bonds with the side chain of LC3B Arg70 to stabilize the interaction (Fig. 4D). The neutral and opposite mutations of Arg70 (R70A and R70E) both decrease the binding affinity between LC3B and FUNDC1 pS_17_ peptide significantly (*K*_*D*_ = 15.75 ± 1.50 μmol/L and 60.98 ± 3.44 μmol/L, respectively) (Fig. 4E and Table 2). We also compared our structure with other LC3B-LIR complexes and found the interaction between LC3B Arg70 and FUNDC1 Glu19 is distinctive (Fig. S1). Taken together, these results imply that the interaction between LC3B Arg70 and FUNDC1 Glu19 is essential in the specific recognition between LC3B and FUNDC1.

### Lys49 senses the phosphorylation of FUNDC1 Ser17, enhancing binding affinity

**Fig. 5:**
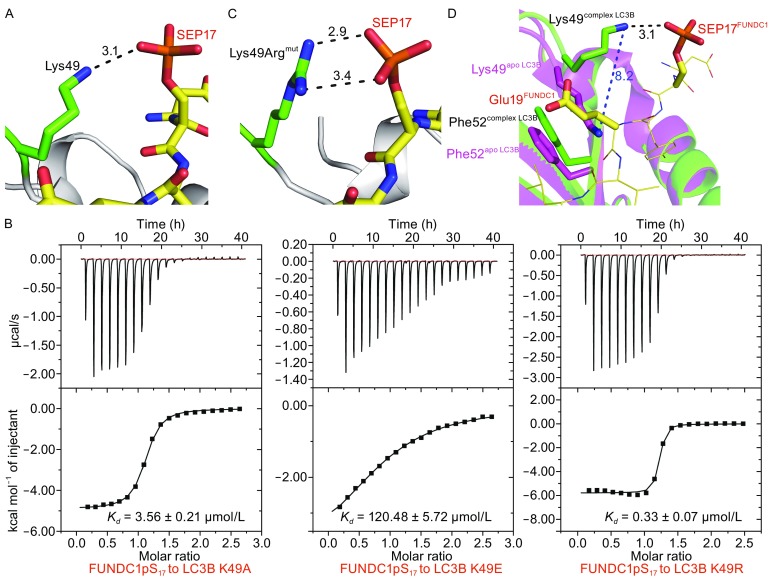
**Molecular basis for the specific recognition between LC3B Lys49 and phosphorylated Ser17 in FUNDC1**. (A) Interaction of LC3B Lys49 (green) with the phosphate group of FUNDC1 SEP17 (orange). The surrounding structure of LC3B is represented in grey. Hydrogen bonds are indicated as black dashes. (B) The ITC fitting results of FUNDC1 pS17 peptide with LC3B mutant at the Lys49 position. (C) The hypothetical model of the interaction between Lys49Arg^mut^ (K49R^mut^ mutated in PyMOL) and the phosphate group of FUNDC1 SEP17. (D) The superimposition of the LC3B-FUNDC1 complex and apo LC3B. The LC3B in complex is colored green and labeled in black while the FUNDC1 pS17 peptide is colored yellow and labeled in red. The apo LC3B is colored and labeled in magenta. The shift of LC3B Lys49 is represented by blue dashes

As we have mentioned previously, ULK1 kinase promotes the phosphorylation of FUNDC1 Ser17, enhancing the interaction with LC3B during mitophagy (Wu et al., 2014). Our ITC experiments also confirmed an enhanced interaction between the FUNDC1 peptide and LC3B by the phosphorylation of FUNDC1 Ser17 (Fig. 1B). Structural analysis shows that the enhancement of binding affinity induced by FUNDC1 pS_17_ is due to an additional hydrogen bond formed between LC3B Lys49 and the phosphate group of FUNDC1 pS_17_ (Fig. 5A). As shown in Fig. 5B, the alanine substitution of LC3B Lys49 (K49A), which prevents the formation of this hydrogen bond, results in a ~6-fold decrease in the binding affinity of LC3B for FUNDC1 pS_17_ peptide (*K*_*D*_ = 3.56 ± 0.21 μmol/L). Furthermore, the phosphorylation of Ser17 leads to an elongated and electronegative side chain, allowing it easily approach the positively charged side chain of Lys49. Mutation of Lys49 to glutamine (K49E) results in a >200-fold reduction in binding affinity (*K*_*D*_ = 120.48 ± 5.72 μmol/L) due to the electrostatic repulsion between two electronegative side chains (Fig. 5B). In contrast, the mutation of Lys49 to arginine (K49R) enhances the binding affinity of LC3B for the FUNDC1 pS_17_ peptide with a *K*_*D*_ value of 0.33 ± 0.07 μmol/L (Fig. 5B). This may be attributed to the additional hydrogen bond(s) formed between the arginine residue and the phosphate group of pS_17_ (Fig. 5C).

Additionally, we compared the conformations of Lys49 in our LC3B-FUNDC1 complex structure and apo LC3B (PDB ID: 3VTU) (Fig. 5D). Interestingly, the side chain of LC3B Lys49 undergoes a large structural rearrangement in two structures. In apo LC3B, the Lys49 side chain forms hydrophobic interactions with the aromatic ring of Phe52, occupying the space for the side chain of the FUNDC1 Glu19 in the complex structure. However, in the complex structure, a largely shift (8.2 Å) of the Lys49 side chain is induced by its interaction with the pS_17_ of FUNDC1, providing enough space to accommodate the FUNDC1 LIR, especially the side chain of Glu19 (Fig. 5D).

Taken together, our mutational and structural analyses of LC3B Lys49 reveal that Lys49 is essential in sensing the phosphorylation state of FUNDC1 Ser17 for selective mitophagy (Table 2).

### HADDOCK models the interface between FUNDC1 Ser13 and LC3B

**Fig. 6:**
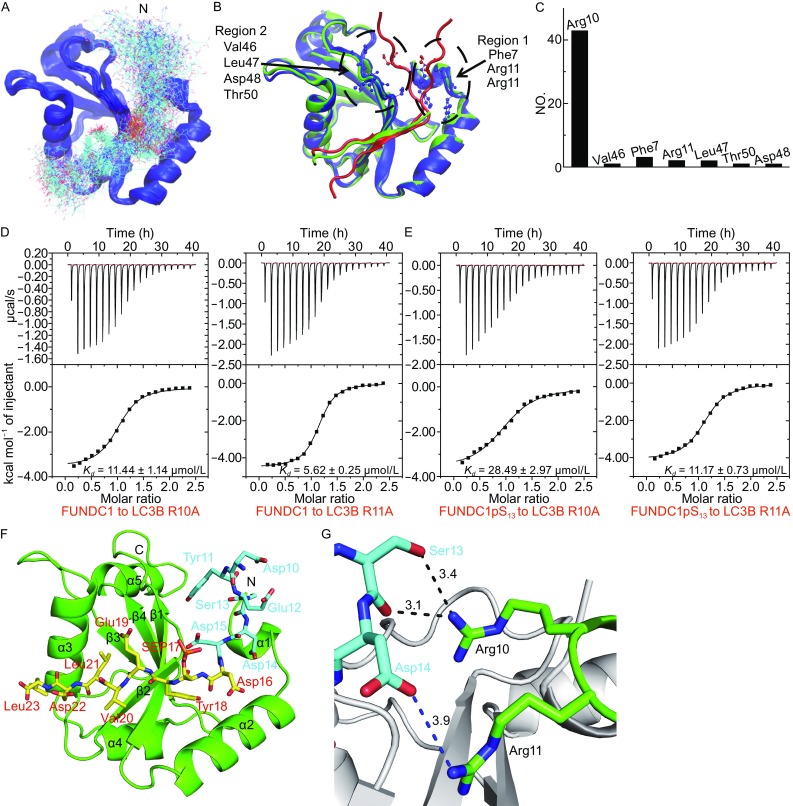
**The HADDOCK model of LC3B-FUNDC1** ^**10–23**^ **complex**. (A) The initial 200 models of the LC3B-FUNDC1^10–23^ complex calculated by HADDOCK. LC3B is colored blue and the FUNDC1 pS_17_ peptide is colored cyan. The N-terminus of the FUNDC1 pS_17_ peptide is marked. (B) The two most possible regions calculated from the statistics of initial HADDOCK models. Our crystal structure is shown in green and the calculated models are colored in blue and red, respectively, and labeled in black. (C) The statistics of the most probable LC3B residues interacting with FUNDC1 Ser13. (D and E) The ITC fitting results of FUNDC1 unphosphorylated peptide and pS_13_ peptide with LC3B mutants at the Arg10 and Arg11 positions. (F) The possible model of the LC3B-FUNDC1^10–23^ complex calculated by HADDOCK. LC3B and FUNDC1 pS_17_ peptide^16–23^ are shown in green and yellow, respectively. The N-terminus of the FUNDC1 pS_17_ peptide^10–15^ is colored and labeled in cyan. (G) The possible interactions of LC3B Arg10 and Arg11 (green) with Ser13 and Asp14 (cyan) of FUNDC1 pS_17_ peptide. Hydrogen bonds and electrostatic interaction are indicated as black and blue dashes, respectively

Unlike Ser17, FUNDC1 Ser13 is dephosphorylated by PGAM5 phosphatase when cells are treated with hypoxia or mitochondrial uncouplers to enhance the interaction with LC3B (Chen et al., 2014), which was confirmed by our ITC assays *in vitro* (Fig. 1B). In our complex structure, the N-terminus of the FUNDC1 pS_17_ peptide^10–15^, including Ser13, was not visible in the electron density map. Therefore, we generated a LC3B-FUNDC1^10–23^ complex model using HADDOCK to study the interface between FUNDC1 Ser13 and LC3B (Fig. 6). Two hundred refined structures were generated by the HADDOCK run, and the N-terminal end of the FUNDC1 peptide was not converged among these structures (Fig. 6A). Hydrogen bonding interactions between residues in the N-terminus of the FUNDC1 peptide and those in LC3B were further analyzed. The structures suggest that the LC3B forms hydrogen bonding interactions with residues in the N-terminus of FUNDC1 in only 53 structures among the 200 structures. The N-terminus of the FUNDC1 peptide orients to the N-terminal end (Region 1) or the linker of β1β2 (Region 2) of LC3B in the 53 structures (Fig. 6B). Among the 53 structures, 43 structures use LC3B Arg10 and FUNDC1 Ser13 to form hydrogen bonding interactions (Fig. 6C).

To confirm the residues of LC3B that interact with FUNDC1 Ser13, we introduced alanine mutations into region 1 and region 2 of LC3B and performed ITC assays to determine the binding affinities between the unphosphorylated FUNDC1 peptide and LC3B mutants. All thermodynamic parameters of the ITC experiments are listed in Table 2. The ITC results show that the alanine substitution of both LC3B Arg10 and Arg11 (R10A and R11A) notably decreased the binding affinities of LC3B for the FUNDC1 peptide by ~6-fold (*K*_*D*_ = 11.44 ± 1.14 μmol/L) and ~3-fold (*K*_*D*_ = 5.62 ± 0.25 μmol/L), respectively (Fig. 6D). This indicates that the two residues play significant roles in the specific recognition of FUNDC1 Ser13, which is consistent with the interaction information suggested by HADDOCK models. In addition, it has been mentioned above that the phosphorylation of FUNDC1 Ser13 caused a ~3-fold reduction in the binding affinity of LC3B for FUNDC1 peptide (Fig. 1B), while the influence of the LC3B R10A mutation on the binding affinity is much stronger than that of FUNDC1 Ser13 phosphorylation. We speculate that the phosphorylation of FUNDC1 Ser13 may generate steric hindrance for LC3B binding, while the LC3B R10A mutation directly destroys the interaction between Arg10 of LC3B and Ser13 or nearby Asp14 of FUNDC1 peptide, leading to the decreased binding affinity. Furthermore, we measured the binding affinities of the LC3B mutants R10A and R11A with the FUNDC1 pS_13_ peptide. As expected, the ITC results suggest a ~2-fold reduction compared with the unphosphorylated peptide (*K*_*D*_ = 28.49 ± 2.97 μmol/L and *K*_*D*_ = 11.17 ± 0.73 μmol/L, respectively, Fig. 6E). A stronger decrease occurred when glutamine was introduced into Arg10 (*K*_*D*_ = 34.01 ± 3.46 μmol/L) and Arg11 (*K*_*D*_ = 17.12 ± 1.05 μmol/L), due to the electrostatic repulsion between the two electronegative side chains (Table 2).

Based on these results, a new complex model was generated using HADDOCK for more detailed insights into the interaction between FUNDC1 Ser13 and LC3B (Fig. 6F). In this model, the N-terminus of the FUNDC1pS_17_ peptide lies on the first α-helix (α1) of LC3B with only a few contacts (Fig. 6F). This implies that the N-terminus of this peptide is flexible, which makes sense, as this region is missing in the electron density map. The side chain of LC3B Arg10 forms hydrogen bonds with the side chain and backbone carbonyl group of FUNDC1 Ser13 (Fig. 6G). Alternatively, LC3B Arg11 interacts with the negatively charged side chain of Asp14 in FUNDC1 (Fig. 6G). All HADDOCK results and ITC analyses show that LC3B Arg10 is the key residue that interacts with FUNDC1 Ser13 whose phosphorylation may generate steric hindrance for Arg10 binding.

## Discussion

FUNDC1, a new receptor for hypoxia-induced mitophagy in mammalian cells, was first reported in 2012 (Liu et al., 2012). Since then, functional studies of the FUNDC1-mediated mitophagy pathway have been extensively reported. Under hypoxia or FCCP stress, FUNDC1 Ser17 is phosphorylated, while Ser13 and Tyr18 are dephosphorylated to enhance the interaction with LC3B and recruit LC3-related autophagosomes to eliminate dysfunctional mitochondria (Liu et al., 2012; Chen et al., 2014; Wu et al., 2014). Thus, post-translational modification, especially phosphorylation, plays an essential role in regulating mitophagy. However, few studies have sufficiently elucidated the phosphorylation impact on the interaction between mitophagy receptors and LC3B from a structural point of view. Moreover, the molecular mechanism of the specific LC3B recognition for FUNDC1 also remains unclear. In this study, we solved the crystal structure of LC3B in complex with the FUNDC1 pS_17_ peptide. The binding interface of the FUNDC1 pS_17_ peptide on LC3B provides the structural elucidation for the specific recognition.

Our structure comparison reveals that, upon the binding with the FUNDC1 pS_17_ peptide, the side chain of LC3B Lys49 undergoes a large structural rearrangement, to accommodate the phosphorylated FUNDC1 peptide. Previous studies have proposed the local conformational change of LC3B when bound with unphosphorylated receptors (Suzuki et al., 2014). However, regarding the interaction with a phosphorylated peptide, the conformational switch of Lys49 in LC3B was revealed for the first time. Suzuki and co-workers presented a similar rearrangement of Lys49 in the LC3A-Atg13 complex (Suzuki et al., 2014). In their structure, Lys49 switches on the hydrophobic interaction surface of LC3A and in turn binds to Val445 of Atg13, with a 6.7 Å shift for Lys49 side chain compared with 8.2 Å in our structure. We speculate that the negative charge of pS_17_ in FUNDC1 induced by phosphorylation may be the reason for the more obvious shift compared with the uncharged side chain of Atg13 Val445. In addition, they demonstrated that the shift of Lys49 side chain is conserved among LC3 homologs in mammals through the structural comparison of several LC3 homologs (Suzuki et al., 2014). This indicates that the LC3 Lys49 not only regulates the binding of LIR as a switch but also takes charge of the specific recognition of post-translational modification of the mitophagy receptors, which is essential for autophagosome formation and the removal of dysfunctional mitochondria.

As mentioned previously, the reversible phosphorylation of FUNDC1 Ser13 and Tyr18 are regulated by different phosphatases and kinases with two different mechanisms, respectively (Liu et al., 2012; Chen et al., 2014). However, Chen et al. confirmed that the reversible phosphorylation of FUNDC1 Ser13 and Tyr18 functionally cooperate to regulate FUNDC1-mediated mitophagy (Chen et al., 2014). Moreover, Wu and co-workers confirmed that Src kinase, which phosphorylates FUNDC1 Tyr18, suppresses phosphorylation of FUNDC1 Ser17 by ULK1 kinase (Wu et al., 2014), providing evidence that ULK1-mediated FUNDC1 pS_17_ may work cooperatively with FUNDC1 Ser13 and Tyr18 during mitophagy. However, how does FUNDC1 respond to different degrees of stress through different states of phosphorylation? Do the conformation and recognition between LC3B and FUNDC1 change under different levels of FUNDC1 phosphorylation? These questions require further research.

Overall, we provide structural insights into the interaction between LC3B and phosphorylated Ser17 and unphosphorylated Ser13 and Tyr18 of FUNDC1, which is the first step to thoroughly explain the molecular mechanism of FUNDC1-dependent mitophagy. Due to the existence of different post-translational modification sites in FUNDC1, further biochemical, structural and cellular experiments should be studied in order to elucidate how cells synergistically regulate these key residues for responding to stress.

## Materials and methods

### Protein expression and purification

A DNA fragment encoding the full-length (125 amino acids) human LC3B was amplified by PCR from the human brain cDNA library and cloned into the pET-28a(+) expression vector (Novagen), contained an N-terminal 6×His tag and a tobacco etch virus (TEV) protease cleavage site. All mutants of LC3B were generated using a MutanBEST kit (Takara) and confirmed by DNA sequencing. The proteins were expressed in *Escherichia coli* BL21 (DE3) cells (Novagen) cultured in LB medium at 37 °C to OD_600_ = 0.8, then shifted to 16 °C and induced with 0.5 mmol/L IPTG overnight. Bacterial pellets were resuspended in buffer A (20 mmol/L Tris-HCl, 1 mol/L NaCl, pH 8.0) and lysed by sonication on ice. Then, soluble proteins were purified with a Ni^2+^-chelating column (GE Healthcare), followed by a Superdex 75 column (GE Healthcare). After being cleaved by TEV protease overnight at 16 °C to remove the 6×His tag, the purified protein was concentrated to ~20 mg/mL in buffer B (20 mmol/L Tris-HCl, 200 mmol/L NaCl, 1 mmol/L EDTA, pH 8.0) and stored at −80 °C.

### Peptide preparations

Peptides were synthesized by GL Biochem (Shanghai), and stock solutions (5 to 15 mmol/L) were prepared in buffer B. The sequences of the peptides are as follows: (p: phosphorylation; pS: phosphorylated Ser; pY: phosphorylated Tyr)

FUNDC1: DYESDDDSYEVLDLTE

FUNDC1 pS_17_: DYESDDD-pS_17_-YEVLDLTE

FUNDC1 pS_13_: DYE-pS_13_-DDDSYEVLDLTE

FUNDC1 pY_18_: DYESDDDS-pY_18_-EVLDLTE

### Isothermal titration calorimetry (ITC)

ITC assays were performed on a MicroCal iTC200 calorimeter (GE Healthcare) at 25 °C. The concentrations of proteins were determined spectrophotometrically. Proteins and peptides were dialyzed against buffer B and adjusted to 0.25 mmol/L and 3 mmol/L, respectively. Curve fitting to a single binding site model was performed by the ITC data analysis module of Origin 7.0 (MicroCal) provided by the manufacturer. The thermodynamic parameters of the ITC experiments are listed in Table 2.

### Crystallization, data collection and structure determination

LC3B^1–125^ and FUNDC1pS_17_ peptide^10–25^, mixed at a 1:2 molar ratio, were crystallized in 30% PEG MME 2000, 0.1 mmol/L sodium cacodylate (pH 6.0) at 16 °C by vapor diffusion in sitting drops. Crystals were soaked in cryoprotectant made of mother liquor supplemented with 20% glycerol before being flash-frozen in liquid nitrogen. Data sets were collected on Beamline 17U at Shanghai Synchrotron Radiation Facility (SSRF). The structure of the LC3B-FUNDC1 complex was solved by molecular replacement with the program MOLREP (Vagin and Teplyakov, 2010), using the apo LC3B^2–123^ (PDB ID: 3VTU) as the search model (Rogov et al., 2013). The FUNDC1 pS_17_^10–25^ peptide was then modeled in COOT (Emsley et al., 2010), and the structure of the LC3B-FUNDC1 complex was refined by the programs REFMAC5 (Murshudov et al., 2011) and PHENIX.refine (Adams et al., 2010). Crystal diffraction data and refinement statistics are displayed in Table 1. Structure analysis was performed using COOT and PyMOL (http://www.pymol.org/).

### Coordinates

Coordinates and structure factors for the LC3B-FUNDC1 pS_17_ peptide complex have been deposited in the Protein Data Bank (PDB) under the accession codes 5GMV.

### Generation of the LC3B-FUNDC1 pS_17_ peptide^10–23^ complex model by HADDOCK

Our crystal structure containing LC3B and the N-terminus truncated FUNDC1 peptide (Δ10–15) was used in model building, and two rounds of docking were performed using the easy interface of the HADDOCK webserver (de Vries et al., 2010). The first round of the docking procedure was performed as follows. First, coordinates of the missing residues of the FUNDC1 peptide were built using PyMOL and Ser17 in the FUNDC1 peptide was phosphorylated by PyTMs (Warnecke et al., 2014). Then, inputs of the HADDOCK webserver were extracted from the crystal structures. Interface residues involved in inter-chain hydrogen bonding interactions were treated as active residues of their corresponding chains. Passive residues were defined automatically around the active residues. Two hundred refined structures were generated by the HADDOCK run, and the N-terminal of the FUNDC1 peptide was not converged among these structures. In the second round of the docking procedure, Arg10 and Arg11 in LC3B and Ser13 in FUNDC1 peptide were included in active residues. The second docking procedure resulted in another 200 refined structures. The 200 refined structures were divided into 5 clusters by the single linkage cluster method with a distance cut-off of 0.2 Å, and 196 structures were involved in cluster 5, representing 98% of the models HADDOCK generated. Then, we chose the representative conformation of cluster 5 as our model.

## Electronic supplementary material

Below is the link to the electronic supplementary material.

Supplementary material 1 (PDF 276 kb)
