# Supplementary material for: Structural insights into the recognition of phosphorylated FUNDC1 by LC3B in mitophagy
Source: Protein Cell. 2016 Oct 18;8(1):25–38. doi: 10.1007/s13238-016-0328-8 (PMC5233613; doi:10.1007/s13238-016-0328-8)
Supplement: Supplementary file 1 — Supplementary material 1 (PDF 276 kb) [file 13238_2016_328_MOESM1_ESM.pdf]

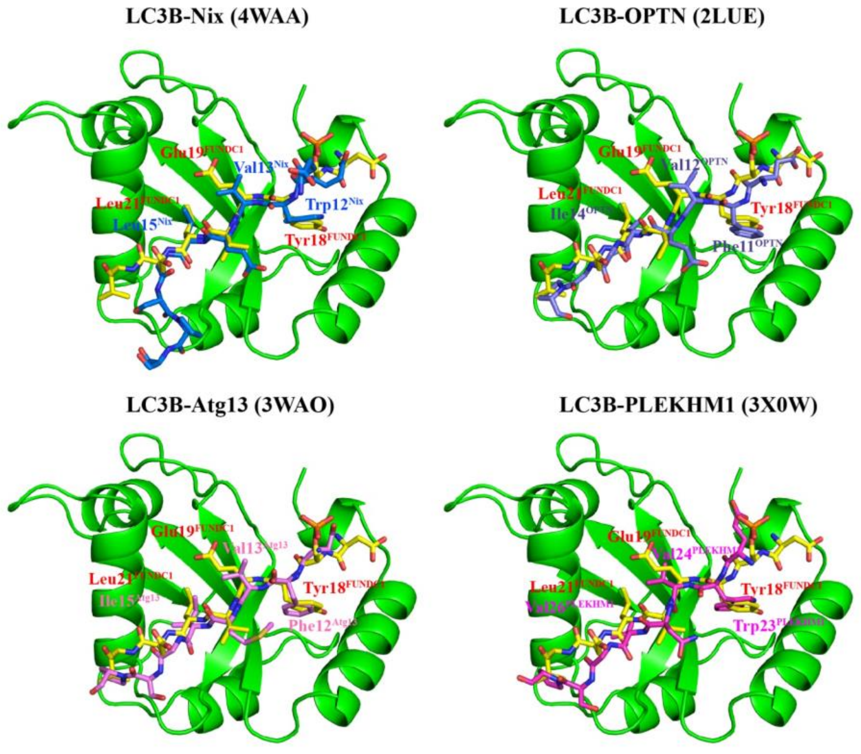

Supplementary Figure S1. Structural Comparisons of LC3B-FUNDC1 complex with other LC3B-LIR complexes. The superimpositions of the LC3B-FUNDC1 complex on LC3B-Nix (PDB code 4WAA), LC3B-OPTN (2LUE), LC3B-ATG13 (3WAO) and LC3B-PLEKHM1 (3X0W), respectively. The superimpositions only show LC3B (green) in the LC3B-FUNDC1 complex (LC3B molecules only display r.m.s. deviations for Cα atoms of 0.600 Å, 0.614 Å, 0.637 Å, 0.585 Å, respectively). The FUNDC1 peptide is shown in yellow and labeled in red, while the Nix, OPTN, Atg13 and PLEKHM1 are shown in marine, slate, pink and magenta, respectively.

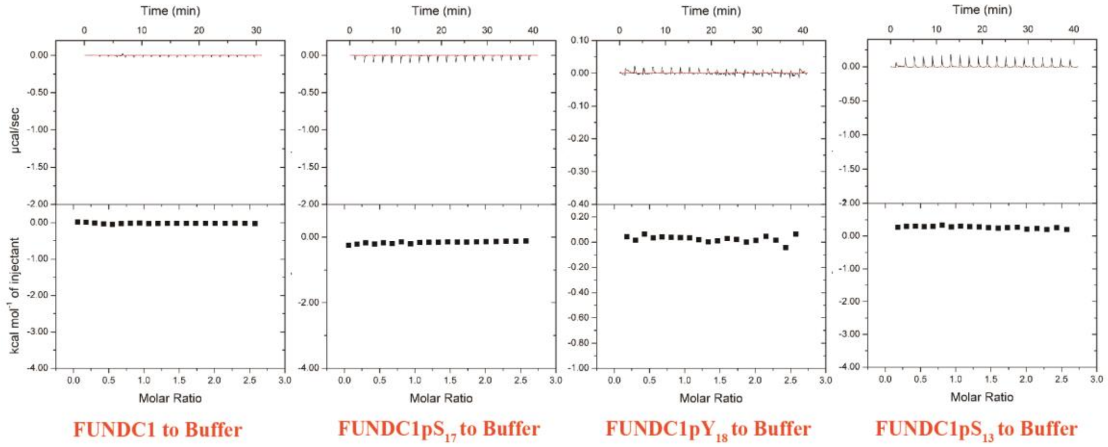

Supplementary Figure S2. The ITC controls of the data in Figure 1B. The ITC control reactions of unphosphorylated FUNDC1 peptide and FUNDC1 peptides phosphorylated at different positions titrated into buffer.
